# When the noise goes on: received sound energy predicts sperm whale responses to both intermittent and continuous navy sonar

**DOI:** 10.1242/jeb.219741

**Published:** 2020-04-06

**Authors:** Saana Isojunno, Paul J. Wensveen, Frans-Peter A. Lam, Petter H. Kvadsheim, Alexander M. von Benda-Beckmann, Lucía M. Martín López, Lars Kleivane, Eilidh M. Siegal, Patrick J. O. Miller

**Affiliations:** 1 Sea Mammal Research Unit, Scottish Oceans Institute, University of St Andrews, St Andrews, Fife KY16 8LB, UK; 2 Faculty of Life and Environmental Sciences, University of Iceland, Askja, Sturlugata 7, 102 Reykjavik, Iceland; 3 Acoustics and Sonar, Netherlands Organization for Applied Scientific Research (TNO), PO Box 96864 The Hague, 2509 JG, The Netherlands; 4 Defence Systems Division, Norwegian Defense Research Establishment (FFI), NO-3191 Horten, Norway; 5 LKARTS-Norway, NO-8290, Skutvik, Norway

**Keywords:** Anthropogenic noise, Continuous active sonar, DTAG, Intermittent sound, Time budget, Time-series model

## Abstract

Anthropogenic noise sources range from intermittent to continuous, with seismic and navy sonar technology moving towards near-continuous transmissions. Continuous active sonar (CAS) may be used at a lower amplitude than traditional pulsed active sonar (PAS), but potentially with greater cumulative sound energy. We conducted at-sea experiments to contrast the effects of navy PAS versus CAS on sperm whale behaviour using animal-attached sound- and movement-recording tags (*n*=16 individuals) in Norway. Changes in foraging effort and proxies for foraging success and cost during sonar and control exposures were assessed while accounting for baseline variation [individual effects, time of day, bathymetry and blackfish (pilot/killer whale) presence] in generalized additive mixed models (GAMMs). We found no reduction in time spent foraging during exposures to medium-level PAS (MPAS) transmitted at the same peak amplitude as CAS. In contrast, we found similar reductions in foraging during CAS (d.f.=1, *F*=8.0, *P*=0.005) and higher amplitude PAS (d.f.=1, *F*=20.8, *P*<0.001) when received at similar energy levels integrated over signal duration. These results provide clear support for sound energy over amplitude as the response driver. We discuss the importance of exposure context and the need to measure cumulative sound energy to account for intermittent versus more continuous sources in noise impact assessments.

## Abstract (precis)

**Summary:** Experimental exposure of sperm whales to continuous and pulsed sonar reveals that total pulse energy predicts cessation of foraging better than pulse amplitude or duty cycle.

## INTRODUCTION

Noise pollution caused by human activities is ubiquitous in terrestrial and aquatic environments around the world, with detrimental effects on both human and animal health and behaviour (Kight and Swaddle, 2011; Shannon et al., 2016; Williams et al., 2015; Faulkner et al., 2018). Anthropogenic sources vary in amplitude, spectral and temporal patterns, ranging from intermittent (pulsed) to more continuous sounds (Hildebrand, 2009; Shannon et al., 2016). Understanding how these different types of sound exposures influence physiology and behaviour is crucial for assessing and mitigating noise pollution impacts.

Sonars have wide-ranging civilian, military and scientific applications (Hildebrand, 2009). Conventional active sonar systems transmit short pulses followed by a longer period of listening for echo returns (pulsed active sonar, PAS). However, recent advances in naval sonar and signal processing technologies allow for simultaneous transmission and listening (continuous active sonar, CAS). This technology allows for greater duty cycle (percentage of time with active transmission) leading to near-continuous illumination of a target and therefore more detection opportunities (van Vossen et al., 2011; Bates et al., 2018). In seismic surveying, high duty cycle sound sources (vibroseis) are being considered as an alternative to impulsive airgun sounds (Duncan et al., 2017). Similar high duty cycle alternatives are being developed for pile-driving activities (Graham et al., 2017). More continuous introduction of energy into the marine environment may allow the use of lower source levels (in terms of peak amplitude), with similar or even greater cumulated exposure levels. This could lead to more severe environmental impact, especially by increasing the risk of auditory masking because more continuous sound transmissions provide fewer silent periods and opportunities for auditory recovery.

Anthropogenic noise can cause animals to trade off fitness-enhancing activities such as foraging or resting, and invest time and energy in behavioural responses such as avoidance. If persistent, such behavioural disturbance might lead to increased population vulnerability (Pirotta et al., 2018). Understanding changes in fitness-enhancing activities and subsequent life functions is crucial for linking the impacts of multiple stressors at an individual level to potential impacts at a population level (NAS, 2017). Marine mammals are expected to be particularly vulnerable as they rely on sound for key life functions and behaviours. Extensive behavioural response studies on the effects of navy sonar have quantified the probability of responses, such as avoidance or cessation of foraging, as a function of received acoustic levels (‘dose–response’ curves) (e.g. Miller et al., 2014; Harris et al., 2015). However, these studies have focused on PAS signals and it remains unclear how such results can be extrapolated to include effects of CAS signals. Several studies have shown differing responses to intermittent versus continuous noise exposures (e.g. fish species: Nichols et al., 2015; Blom et al., 2019).

Sperm whales (*Physeter macrocephalus* Linneaus 1758) are considered to be medium-sensitive to navy sonar in terms of their behavioural responsiveness (Harris et al., 2015) and studies in northern Norway have documented reduced foraging effort in response to pulsed 1–2 kHz sonar (Isojunno et al., 2016). Similar responses were observed following playbacks of killer whale (potential predator) sounds (Curé et al., 2016; Isojunno et al., 2016). Sperm whales found in these high-latitude foraging grounds are thought to typically be solitary foraging males, while females remain at lower-latitude breeding grounds (Best, 1979; Teloni et al., 2008). Animal-attached acoustic- and movement-recording tags (DTAG; Johnson and Tyack, 2003) allow monitoring of sperm whale echolocation clicks, including ‘buzzes’ as an indicator of prey capture attempts (Miller et al., 2004a), and classification of distinct functional behaviours, including foraging, resting (Miller et al., 2008) and potential disturbance states (e.g. active non-foraging state; Isojunno and Miller, 2015; Isojunno et al., 2016).

The objectives of this study were to quantify any differences in behavioural responses to CAS versus PAS, and contrast what aspects of the exposure conditions (peak signal amplitude, sound energy or duty cycle) best predict response intensity while controlling for contextual and environmental variability (e.g. water depth, presence of other cetaceans). Response intensity was defined as the probability and duration of change in foraging effort, i.e. time spent in foraging versus non-foraging behaviours (time budget), and given the time budget, proxies for foraging success or locomotion costs (Isojunno and Miller, 2015). Experimental PAS was transmitted at a source level matching either CAS peak amplitude (medium-level PAS, MPAS) or total pulse energy (high-level PAS, HPAS). We hypothesized that if peak signal amplitude was the response driver, HPAS should elicit greater response intensity than MPAS and CAS. Alternatively, if signal energy was the main driver, we expected similar responses to HPAS and CAS. If the signal duty cycle was the driver of disturbance, we expected CAS to elicit the greatest response intensity (Table 1).

**Table 1.**
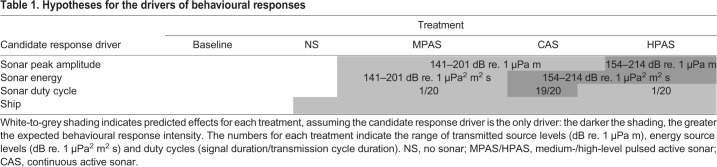
**Hypotheses for the drivers of behavioural** **responses**

## MATERIALS AND METHODS

### Use of animals in research

Animal experiments were carried out under permits issued by the Norwegian Animal Research Authority, in compliance with the ethical use of animals in experimentation. The research protocol was approved by the University of St Andrews Animal Welfare and Ethics Committee.

### Field data collection

Data were collected for sperm whales in 2016–2017 in the Norwegian Sea north and west off Andenes, Norway (Lam et al., 2018a,b). Sperm whales were located visually and acoustically by monitoring their echolocation clicks with a hydrophone array towed by the 55 m research vessel R/V *H.U. Sverdrup II*. Audio- and movement-recording data loggers (DTAGs; Johnson and Tyack, 2003) were deployed from a small tag boat and attached to the whale using suction cups. The research vessel aimed to sail 4.3 km (2.3 nautical miles) wide square boxes around the tagged whale to facilitate acoustic and visual tracking. When at the surface, the tagged whale was tracked visually and using VHF transmissions from the tag.

### Experimental design

The baseline period was considered to have begun once the tag boat left the tagged whale (Isojunno and Miller, 2015) and lasted at least 4 h. The subsequent experimental phase consisted of a sequence of approaches by the source vessel (‘exposure sessions’), each lasting 40 min and with a minimum of 1 h 20 min between sessions. The vessel was positioned to approach the focal whale from 4 nautical miles (7.4 km) distance at an angle of 45 deg to the expected path of the whale while towing the sonar source (SOCRATES, TNO, The Netherlands) at an average depth of 55 m (range 35–100 m). Approach speed (8 knots=4.1 m s^−1^) and course were kept constant throughout each session. The sonar source was towed but not transmitting during no-sonar control approaches, which were always conducted first in the sequence.

Each exposure session in the sequence consisted of one of three possible sonar transmission schedules, presented in a rotating order (Lam et al., 2018a,b): (1) HPAS, 1 s hyperbolic upsweep from 1 to 2 kHz with a maximum source level of 214 dB re. 1 μPa m; (2) MPAS, 1 s hyperbolic upsweep from 1 to 2 kHz with a maximum source level of 201 dB re. 1 μPa m; or (3) CAS, 19 s hyperbolic upsweep from 1 to 2 kHz, with a maximum source level of 201 dB re. 1 μPa m (same as MPAS) and an energy source level of 214 dB re. 1 µPa^2^ m^2^ s (same as HPAS). Each signal was transmitted every 20 s, resulting in 5% and 95% duty cycles for PAS and CAS, respectively. Source levels were increased by 60 dB, in 1 dB steps, over the first 20 min of the exposure session (‘ramp-up’). At full power, these signals are representative of operational PAS and potential future CAS use, although operational source levels may be greater in some exercise scenarios.

Sound exposures were conducted at least 20 nautical miles distance from previous exposures within 24 h. Sperm whales in the study area are thought to be mostly solitary (e.g. Teloni et al., 2008). Photo identification was used to check that tags were not repeatedly deployed on the same individuals.

### Data processing

Movement sensor data from the tag were decimated to 5 Hz, and used to calculate depth, acceleration and body pitch angle of the whale using established methods (Johnson et al., 2009; Miller et al., 2004a,b, 2011). To generate a lower resolution time series for behaviour state classification, depth data were downsampled and pitch data were averaged over 1 min intervals to filter out high-frequency movements such as fluking, but to still allow sufficient time resolution to capture surface intervals. Fluke stroke rate was calculated using an automated detector based on cyclic variation in pitch (Johnson and Tyack, 2003; Tyack et al., 2006), with detection parameters determined manually for each tag record by inspecting the magnitude of the stroke signals within the pitch record. Audio data (stereo, sampled at 96 kHz) were monitored aurally and visually using spectrograms to identify acoustic foraging cues, i.e. echolocation click trains, other tagged whale sounds (such as slow clicks and codas with likely social function) and any environmental sounds (other species, anthropogenic noise sources). Rapid increases in click rate (terminal echolocation ‘buzzes’) were used to indicate prey capture attempts (Miller et al., 2004a,b). The presence or absence of prey capture attempts within each 1 min interval was scored using the start time of buzzes.

Killer whales (*Orcinus orca*; OO) and long-finned pilot whales (*Globicephala melas*; GM) were detected regularly during the 2016–2017 field trials. We scored their presence to evaluate whether it might have influenced the behaviour of tagged sperm whales. From acoustic detections alone, it was not always possible to distinguish the two species; their presence was therefore pooled as blackfish events (GMOO). Visual or acoustic detections (on the DTAG or the towed array) of either species within 15 min of each other were defined to be part of the same blackfish event.

In addition, sonar signals other than those involved in the field experiments were detected both in the towed array and on the tags. These ‘unidentified pulsed active sonar’ (UPAS) signals were also defined to be part of the same event when they occurred within 15 min of each other.

The data were then classified into six behaviour states at 1 min time resolution, using a state-switching model in a Bayesian framework (Isojunno and Miller, 2015; Isojunno et al., 2016). The states included: (1) surfacing; (2) descending transit to a deeper depth; (3) layer-restricted search (LRS), searching at a prey layer; (4) ascending transit to a shallower depth or the surface; (5) underwater drifting or resting; and (6) other non-foraging (NF) active state, which could encompass multiple functional behaviours such as socializing or behavioural disturbance (Isojunno et al., 2016). The model structure included a state-specific random walk for depth, probability of echolocation (including both regular and terminal buzz clicks) and state-specific relationships between pitch and vertical speed. Informative priors were used to incorporate biological information (descent and ascent speed, vertical posture during resting, and higher probability of echolocation during foraging). In addition to the 16 tag deployments presented here, 12 additional sperm whale tags from Isojunno et al. (2016) were included in the fitting of the hidden state model. This was done to maximize the data informing the behaviour classification, and also as a consistency check with previous years' behaviour state analysis. The model structure and Bayesian estimation procedure are described in detail in Isojunno and Miller (2015).

Received maximum sound pressure level (SPL) over a 200 ms sliding window (SPL_sp_; dB re. 1 μPa) and sound exposure level integrated over signal duration (SEL_sp_; dB re. 1 μPa^2^ s) were measured in the 0.89–2.24 kHz band for each transmission in the DTAG acoustic recording (Table 2) (Miller et al., 2011).

**Table 2.**
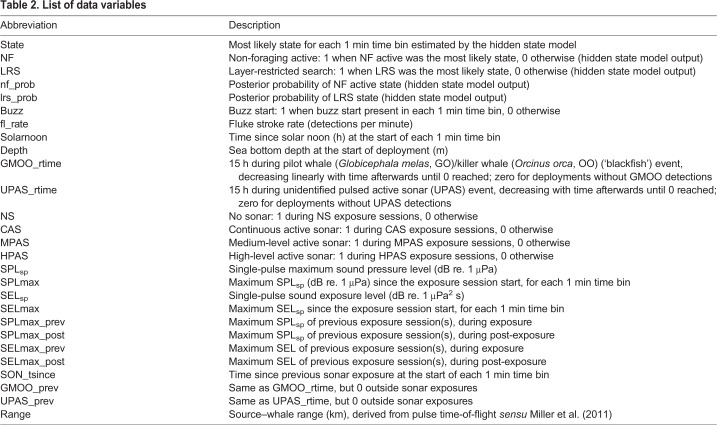
**List of data variables**

### Statistical analysis

Four response variables were considered: probability of (1) NF active (potential disturbance) and (2) LRS behaviour (nf_prob and lrs_prob, respectively), estimated from the posterior of the hidden state-switching model, (3) presence/absence of terminal echolocation clicks (buzz) as a proxy of prey capture attempts and (4) fluke stroke rate (fl_rate) as a proxy of locomotion activity. Each variable was modelled at a 1 min time resolution.

Generalized additive mixed models (GAMMs) were fitted to allow for flexible relationships between the response variables and explanatory covariates (package ‘mgcv’ in R v3.5.1; Wood, 2004). Candidate covariates included both baseline variables (such as time of day, presence/absence and previous exposure to blackfish), variables describing the experimental treatments (CAS, MPAS, HPAS) and received acoustic levels (Table 2). Maximum SPL_sp_ and SEL_sp_ were calculated for the 1 min time series since the start of each sound exposure session and are denoted SPLmax and SELmax, respectively. Order effects and post-exposure effects were included in the analysis in terms of previous maximum SEL or SPL (Appendix Table A1). Covariate selection consisted of two steps. Baseline model selection was carried out first using baseline and post-exposure data alone (with an additional 20 min excluded following each exposure session). The best model was then carried forward for the selection of experimental covariates.

Each tag deployment was fitted as a random effect, and serial correlation was modelled using first-order autoregressive correlation structure. Non-focal (i.e. simultaneously tagged animals that were not the primary subject of the experiment; Table 3) data from during sound exposures were excluded from the GAMM analysis. Please see Appendix 1 for further details.

**Table 3.**
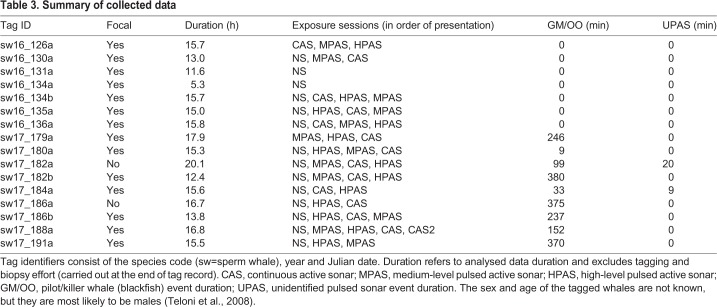
**Summary of collected data**

## RESULTS

### Dataset

A total of 236 h of tag data were analysed from 16 different whales (Table 3). Ten whales were exposed to both types of pulsed sonar (MPAS and HPAS) and continuous sonar (CAS). NS control approaches were conducted for 12 focal whales. All 2017 tag deployments (*n*=9) included blackfish (GMOO) detections, with a total of 32 h of the data considered to be part of blackfish events (visual or acoustic detections occurred within ±15 min). Blackfish events overlapped with 8 different exposure sessions (1 NS, 3 CAS, 3 HPAS and 2 MPAS). Two tagged whales were incidentally exposed to different UPAS from distant naval operations, with a total event duration of 29 min (overlapping with 1 NS and 1 HPAS). These UPAS consisted of tonal signals in different frequency ranges (1.3–3, 3–4, 5, 8 and 10–12 kHz).

**Fig. 1.**
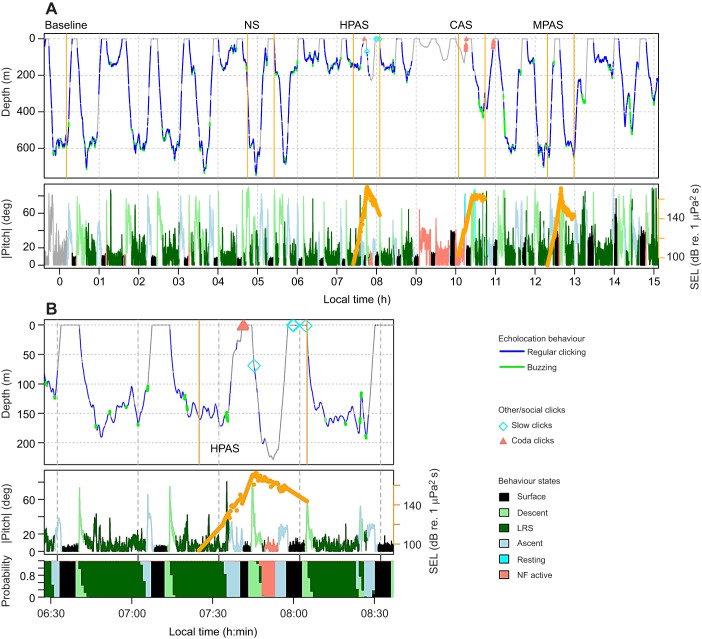
**Example time series.** (A) Full time series for tag deployment sw16_135a. (B) Zoomed-in version of the high-level pulsed active sonar (HPAS) exposure. Tagged whale echolocation and other click production are shown on the dive profile. Orange dots show single-pulse sound exposure level (SEL_sp_) of each received sonar signal. The posterior probability of each state is shown in the bottom panel. Depth and pitch time series are shown at 5 Hz sample rate, states at 1 min time resolution (grey: excluded data). NS, no sonar; CAS, continuous active sonar; MPAS, medium-level pulsed active sonar; LRS, layer-restricted search; NF, non-foraging.

**Fig. 2.**
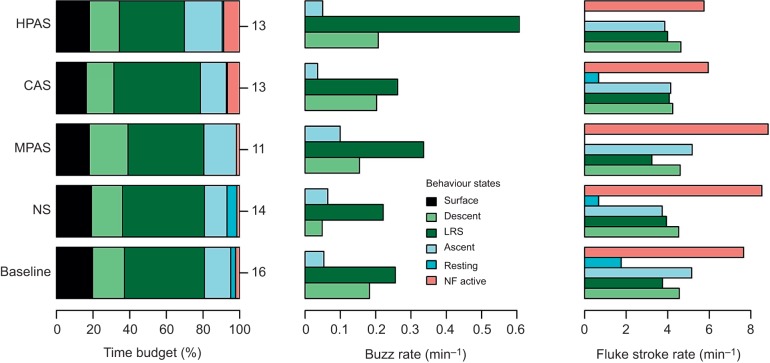
**Individual-average time budget, buzz rate and fluke stroke rate.** Sample sizes (number of individuals) are indicated to the right of the time budgets. Presented data exclude non-focal exposures, 20 min post-exposure periods, and data from during UPAS (unidentified pulsed active sonar) and GM/OO (pilot/killer whale or ‘blackfish’) events. On average during pre-exposure baseline, individuals spent 20% of their time resting at the surface, 17% in descent, 44% in layer-restricted search (LRS) state, 14% on ascent, 2.7% resting or drifting and underwater, and 2.3% in NF active behaviour. Buzzes were produced at an individual-average rate of 0.18 min^−1^ during descent, 0.26 min^−1^ during the LRS state and 0.05 min^−1^ during ascent. Individual average fluke stroke rates were 4.6 min^−1^ during descent, 3.7 min^−1^ during the LRS state and 5.2 min^−1^ during ascent. During baseline, the highest fluke stroke rates were during the NF active state (7.7 min^−1^).

The hidden state-switching model estimated most of the time series to be behaviour states related to foraging dives, with less than 3% of time spent in the non-foraging active state during pre-exposure baseline (Figs 1 and 2; Fig. S1). Fourteen animals switched to non-foraging active state following sound exposure (individual-average SELmax at onset 143 dB re. 1 μPa^2^ s, *n*=26; Fig. S2). The most likely behaviour state continued to be non-foraging active for an average of 1.4 min during MPAS (*n*=6, maximum 4 min), 2.25 min during CAS (*n*=6, maximum 9 min) and 3.8 min during HPAS (*n*=8, maximum 18 min). Its average duration during baseline was 1.9 min (*n*=75, maximum 15 min).

### Behavioural response analysis

The covariate selection procedure supported the following final models (see Appendix 2 for detailed results and Table 2 for variable definitions): (i) P(NF active state)∼s(solarnoon)+s(SELmax); (ii) buzz presence/absence (min^−1^)∼LRS+s(SELmax_prev)+s(SEL_post); and (iii) fluke stroke rate (min^−1^)∼state+s(solarnoon)+s(SELmax_prev).

No baseline or exposure covariates were supported in models with P(LRS) as the response variable. Acoustic dose metrics were only supported in models for the NF active state. Order effects (e.g. SELmax_prev) were retained in the model selection for buzz presence and number of fluke strokes, but not immediate effects of sound exposure (e.g. SELmax, CAS). There was no support for effects of NS approach or incidental sonar in any of the models.

**Fig. 3.**
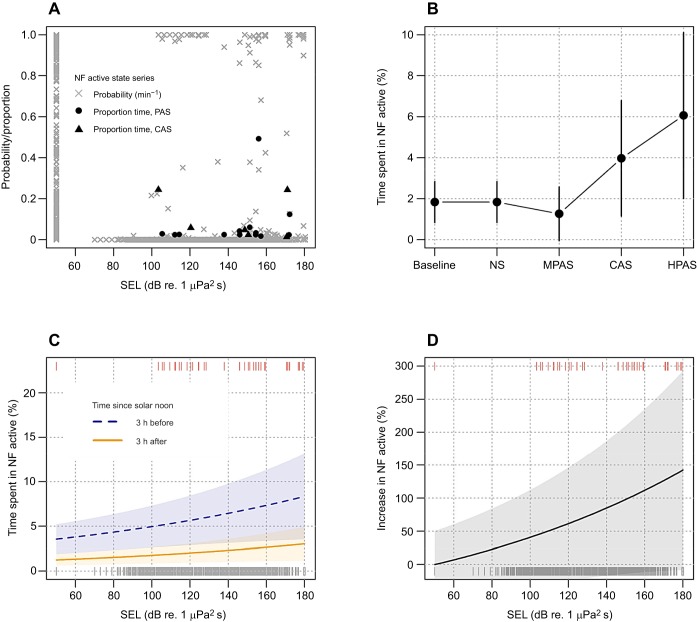
**Non-foraging (NF) active state behaviour during sound exposures.** (A–D) State-switching model output for NF active behaviour (A) was used as response data for generalized additive mixed models (GAMMs) that included the experimental exposure effects (B: exposure session model, C,D: final exposure model). Proportions of time in NF active in each session are shown for SEL_max_ values at the first switch to this behaviour state (A). GAMM estimates in B are given with 95% confidence intervals. Estimates in C are given with time since solar noon fixed to midday. D shows final exposure model estimates as percentage increase from baseline.

CAS and HPAS were clearly supported as predictors for increased time in the NF active state (Wald test, d.f.=1, CAS: *F*=8.0, *P*=0.005; HPAS: *F*=20.8, *P*<0.001) while NS and MPAS were not. Time spent in the NF active state was estimated to increase by a factor of 2.2 during CAS and 3.4 during HPAS (Fig. 3B).

Depending on the time of day, the individual-average time spent in NF active behaviour was estimated to increase from baseline (1.3–3.6%; range 3 h after – 3 h before solar noon) to 3.0–8.3% during exposures when SEL_sp_ exceeded 180 dB re. 1 μPa^2^ s (Fig. 3C), representing >100% increase in time spent in this behaviour state (Fig. 3D). Time spent in the NF active state peaked twice a day, 3 h before and 9 h after solar noon (Fig. S3).

Following sonar exposure sessions with a SEL_sp_ of 180 dB re. 1 μPa^2^ s, the expected value for buzz presence for LRS states decreased from 20.7% (95% CI 15.5–25.8) to 15.1% (10.8–19.4) during post-exposure and 13.6% (8.7–18.6) during subsequent exposures (Fig. S4). Similarly, following sonar exposure sessions with a greater SEL_sp_, fluke stroke rates were reduced during the subsequent sonar exposures (Fig. S5). However, neither model provided a good fit to the data (Fig. S6).

Despite an apparent increase in NF active behaviour within 3 h of a blackfish (GMOO) event (average 3.8 h, maximum 15 h; Fig. S1), there was no clear statistical support for the effect of GMOO or GMOO_rtime (Appendix 2). There was some support for GMOO_prev in models for the NF active state, indicating that whales were more likely to switch to the NF active state during sonar exposures following blackfish events. However, the final model with SEL provided a better fit to the data during sonar exposures than the model with the blackfish sensitization effect (Appendix 2).

## DISCUSSION

Our experiments were designed to contrast the effect of pulsed versus continuous active sonar (PAS versus CAS) on sperm whale behaviour, and compare peak signal amplitude versus energy (SPL versus SEL) as well as duty cycle as response predictors. Our data clearly supported the hypothesis that sound energy was the main driver (Table 1), with similar foraging reductions during CAS and HPAS sonar treatments. No effect of MPAS was found, even though it was transmitted at the same source level amplitude as CAS. Thus, the higher SEL led to stronger responses for sonar received at the same SPL, while we found no evidence for different responses to CAS versus PAS when received at similar SELs.

These results highlight the importance of accounting for signal duration in impact assessments. In particular, only using SPL as a sound exposure metric to predict responses may underestimate impacts of more continuous exposures. While more data are being collected on the specific impacts of CAS systems, it is possible to calculate expected response intensity to CAS based upon existing dose–response curves for PAS using SEL to account for the greater signal duration and/or duty cycle. Our statistical results were robust to the choice of time window used to cumulate SEL (from single to all pulses within a 40 min session). However, we caution against extrapolating our results to exposure durations that are significantly longer than those tested here.

Studies that have systematically compared responses to intermittent versus continuous sound exposures have led to different conclusions about their relative effects in different species (humans: Dornic and Laaksonen, 1989; Pirrera et al., 2010; fish: Nichols et al., 2015; Blom et al., 2019; harbour porpoise: Kok et al., 2018). This might reflect different underlying factors or sets of factors driving the response, such as annoyance, distraction, anti-predator responses, novelty of the sound or masking potential. Also, intermittent sounds varied between studies, with repetition times ranging from seconds to hours. Further studies are required to better understand the underlying mechanisms of cetacean responses to CAS and PAS.

For sperm whales, these results indicated substantially lower response intensity to pulsed 1–2 kHz sonar than previous research in the same area (Isojunno et al., 2016). Context factors that could have changed from previous years include habitat variables, such as potential changes in the prey field and blackfish (pilot or killer whale) presence, prevalence of 1–2 kHz sonar in the environment, or minor differences in the experimental protocol. In the previous study, a faster ramp-up was used and a separate observation vessel from the source vessel allowed adjustments of the course of the source vessel in the initial phase of the approach. The Norwegian Navy introduced 1–2 kHz towed array sonar into service in 2006–2011, which was their first operational sonar in this lower frequency band. Therefore, 1–2 kHz sonar may have been a more novel presentation to the animals in this habitat in 2008–2009 compared with 2016–2017. Novelty of the sonar stimulus was also suggested as an important response driver for northern bottlenose whales (*Hyperoodon ampullatus*), another deep-diving cetacean species (Wensveen et al., 2019).

We aimed to account for variations in environmental and exposure context by including multiple candidate covariates in the analysis, such as habitat depth, time of day and order effects (Table 2). Time of day in particular was supported as a predictor of time spent non-foraging and fluke stroke rates in each behaviour state. Nevertheless, significant variation across tag records in response intensity also remained, indicating that we did not capture all individual variation and/or context variables. Other important context variables could include individual factors (age, body condition, experience) and environmental variation, such as resource quality or distribution (Beale, 2007; Harris et al., 2017).

One context variable that we did test was whether the presence of blackfish, potential predators and/or competitors for sperm whales, influenced their baseline behaviour or responsiveness to sonar. While we found no clear statistical support for a change in baseline behaviour, those whales that were previously exposed to blackfish were more likely to switch to the NF active state during sonar exposures. The presence/absence of blackfish and time since exposure to blackfish were evenly distributed across the different experiments, reducing concern that they influenced our study results. Nevertheless, heterospecific context as a potential mediator of cetacean responsiveness to anthropogenic disturbance warrants further study, by examining more specific indicators of anti-predator behaviour (e.g. grouping behaviour and social sound production), and conducting further experiments with dedicated sequences of sonar and pilot/killer whale sounds (Curé et al., 2016).

The experimental design presented here could be applied in other study systems to investigate the effects of intermittent versus continuous noise on wildlife, such as continuous alternatives to airgun sounds (Duncan et al., 2017). The source approach geometry and ramp-up protocol enables the escalation of acoustic exposure, which can then be measured and associated with changes in behaviour using high-resolution animal-attached tags to quantify response thresholds. The novelty of our study is the matched design directly contrasting the effects of intermittent versus more continuous sonar sound. This design allowed us to quantify evidence for the candidate response drivers (SEL, SPL or duty cycle) and conclude that SEL was the best predictor of sperm whale behavioural responses to 1–2 kHz sonar.

## Supplementary Material

Supplementary information

## Figures and Tables

**Table A1.**
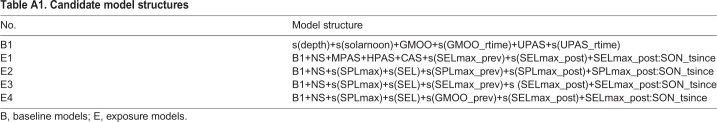
**Candidate model structures**

## Appendix 1

### Statistical modelling methods

#### Statistical model specification

The following steps were taken to reduce correlation in the response variables of the generalized additive models and the chance of testing the same behavioural changes multiple times. Models for posterior probability for layer-restricted search (lrs_prob) and presence/absence of buzz excluded data from non-foraging behaviour states (i.e. surface, resting and non-foraging active state). Also, data from surface periods were excluded from the models for fluke stroke rate as detection of fluke strokes is highly unreliable near the surface. In addition, presence/absence of layer-restricted search was included as a baseline covariate in the models for buzz presence. Similarly, behaviour state was included as a factor covariate in models for fluke stroke rate (fl_rate).

Variables nf_prob, lrs_prob and buzz were specified to follow a binomial distribution (with logit link), whereas fl_rate was specified a Poisson distribution (with log-link). Dispersion parameters were estimated rather than fixed (option ‘quasi’ family) to account for any over- or under-dispersion in the response data.

Sea bottom depth and all received acoustic level metrics were fitted as smooth covariates (thin plate spline). Monotonous increase/decrease was expected for these relationships and therefore the smooth complexity was limited by setting the maximum number of knots to 5. To test for any sensitization/habituation, the maximum received level achieved during the previous exposure session(s) (SELmax_prev, SPLmax_prev; Table 2) was fitted as a smooth covariate in the same way. Time of day was fitted as cyclic smooth (maximum number of knots set to 7). Furthermore, an interaction term between time since previous exposure and received level of that exposure was fitted as a log-linear covariate to test for any post-exposure effects that lasted beyond the exposure end.

The baseline covariates included time since solar noon (solarnoon), water depth (depth), presence/absence of blackfish (GMOO), presence/absence of unidentified pulsed active sonar (UPAS), and time to recovery from blackfish and UPAS events (GMOO_rtime and UPAS_rtime, respectively) (Table 2). GMOO_rtime and UPAS_rtime were used instead of the time since the events so that the covariates would have zero values for deployments without previous GMOO/UPAS events. GMOO_rtime and UPAS_rtime were specified as 15 h (=typical deployment duration) during the events, and decreased with time afterwards, until zero was reached.

#### Covariate selection

Shrinkage smoothers were used as an automatic procedure to reduce model complexity by excluding smooth covariates from each model. Covariates that could not be fitted as shrinkage smooths (presence/absence covariates; Table 2) were removed from the model based on Wald-like tests [using function anova.gam() in mgcv, based on a Bayesian covariance matrix for the coefficients].

The covariate selection was carried out in two steps. First, a full model including all the baseline covariates was fitted to baseline and post-exposure data alone (with an additional 20 min excluded following each exposure session) (Table A1, model B1). A reduced model was then carried forward for the selection of experimental covariates.

For the exposure model selection, four different full models were fitted (E1–E4). All four models included the selected baseline covariates and presence/absence of a no-sonar control approach (NS) (Table A1). The ‘exposure session’ model (E1, Table A1) included presence/absence covariates for each exposure session type (CAS, MPAS, HPAS), to test the alternative hypotheses for the response driver (MS, Table 1). The ‘acoustic dose’ models (E2 and E3, Table A1) included the two received acoustic level metrics [s(SPLmax) and s(SELmax)]. All of the first three exposure models (E1–E3) also included order effects and post-exposure effects, in terms of previous maximum SEL (SELmax_prev, SEL_post) or SPL (SPLmax_prev, SPL_post). The fourth model (E4) included GMOO_prev (time to recovery from blackfish event, but zero outside sonar exposures) instead of the order effect.

If the model did not converge (without the quasi specification), presence/absence covariates were removed. If the model still did not converge, it was assumed to be a poor fit to the data.

## Appendix 2

### Model selection results

#### Non-foraging active state

In the full baseline model for the probability of non-foraging active state (nf_prob), time since solar noon was clearly supported by the Wald test (edf=4.6, *F*=12, *P*<0.001). Also, GMOO_rtime was supported (edf=1.0, *F*=1.3, *P*<0.001); however, the negative *r*^2^ value reported by the model summary indicated a poor model fit. Comparing two baseline models with and without that covariate nf_prob∼s(solarnoon)+s(GMOO_rtime) versus the baseline model: nf_prob∼s(solarnoon), the model excluding GMOO_rtime predicted greater values (2.7% versus 2.3%) when the posterior proportion was above a threshold value (0.9), and lower values when nf_prob was below the threshold (1.8% versus 2.1%), indicating a better model fit. The result was not sensitive to the exact value of the threshold. Therefore, only solarnoon was selected in the final baseline model for non-foraging active state. Almost no serial correlation remained in the residuals of this model.

In the acoustic dose models for nf_prob, SELmax (edf=0.94, *F*=3.6, *P*<0.001) outperformed SPLmax, which was removed by the shrinkage smooth in both models (models E2 and E3). In the experimental session model (E1), Wald tests supported HPAS (d.f.=1, *F*=20.8, *P*<0.001) and CAS (d.f.=1, *F*=8.0, *P*=0.005), but not MPAS (d.f.=1, *F*=0.58, *P*=0.44). In the blackfish model for nf_prob without s(SELmax_post), GMOO_prev was supported (edf=0.98, *F*=5.1, *P*<0.001) while both acoustic dose metrics were removed from the model by the shrinkage smoothing. No-sonar and post-exposure effects were not supported by the Wald test at the 5% level in any of the models for non-foraging. The receiver operating characteristics (ROC) curves from these two models showed a very similar fit to the data.

Two alternative exposure models were therefore considered: nf_prob∼solarnoon+s(SELmax) and nf_prob∼solarnoon+GMOO_prev. They provided a very similar fit to the data. When the model nf_prob∼s(solarnoon)+s(SELmax)+s(GMOO_prev) was only fitted to data including experimental sessions, SEL was supported (edf=0.93, *F*=2.0, *P*=0.002) instead of GMOO_prev (edf=0.47, *F*=0.1, *P*=0.17). Furthermore, the SEL thresholds at which non-foraging behaviour was observed appeared unaffected by previous exposure to blackfish. To infer behaviour responses, therefore, only SELmax was included in the final exposure model for nf_prob. The presence/absence of blackfish and time since exposure to blackfish were evenly distributed across the different experimental exposure sessions. Eight exposures in total overlapped with blackfish events (1 NS, 3 CAS, 3 HPAS and 2 MPAS). There was no statistical evidence for a difference in the distribution of the values at the beginning of the different exposure types (CAS, HPAS or MPAS, *n*=14 when excluding zeros), indicated by Wilcoxon rank sum test (CAS versus other; *W*=22, *P*-value=0.8, *n*=14; HPAS versus other, *W*=25, *P*=0.95; MPAS versus other, *W*=17, *P*=0.73). Therefore, blackfish exposure was unlikely to have influenced the support for SEL over SPL as the dose metric.

#### Layer-restricted search state

The full baseline model for the layer-restricted search (LRS) did not converge unless UPAS was removed. No baseline covariates were supported in the resulting model at the 5% significance level. None of the exposure models for lrs_prob converged with the quasi-specification. With the dispersion parameter fixed to one instead, model E1 and the acoustic dose model E2 did converge; however, no covariates were supported by the Wald tests at the 5% significance level.

#### Buzz presence

For buzz presence, the full baseline model did not converge unless UPAS was removed as a covariate. In the resulting model, presence/absence of LRS was supported (d.f.=1, Wald test, *F*=120, *P*<0.001), as well as GMOO_rtime (edf=0.9, *F*=1.2, *P*<0.001). However, similar to the model for non-foraging active state, the model had a negative adjusted *r*^2^ value, and including GMOO_rtime decreased the model's ability to predict the presence/absence of buzzes. Therefore, only LRS was included in the final baseline model for buzz presence. Some serial correlation remained in the residuals of this model.

For buzz presence, all other exposure models converged except the blackfish model, which only converged with the dispersion parameter fixed to one. Sonar order effects were supported in all models that included them (E1–E3). There was more support for SELmax_prev (edf=0.85, *F*=1.4, *P*=0.005; model E3) than SPLmax_prev (edf=0.80, *F*=1.0, *P*=0.015; model E2). While there was statistical support for SELmax_post (edf=0.86, *F*=1.6, *P*=0.007) and SPLmax_post (edf=0.87, *F*=1.7, *P*=0.005) in the acoustic dose models, there was no support for the SELmax_post:SON_tsince (d.f.=1, *F*=1.0, *P*=0.32) and SPLmax_post:SON_tsince (d.f.=1, *F*=0.76, *P*=0.39) interactions, indicating a constant rather than a decaying post-exposure effect. Similarly, in the exposure session model (E1), only the order effect SELmax_prev (edf=0.78, *F*=1.0, *P*=0.022) and post-exposure effect SELmax_post (edf=0.86, *F*=1.5, *P*=0.008) were supported. The presence/absence covariates for the sonar treatments (NS, CAS, HPAS or MPAS) were not supported at the 5% level. In the blackfish model (E4) which did not include the order effects, SPLmax gained support instead (edf=0.9, *F*=2.26, *P*=0.002) while SEL and GMOO_prev were removed from the model by the shrinkage smoother. Therefore, the final exposure model for buzz presence included LRS, SELmax_prev and SELmax_post. The model fit of the final exposure model for buzz presence was assessed using a ROC curve. The final exposure model estimated a decrease in buzz presence during exposure and post-exposure periods following a greater SEL exposure (Fig. S4).

#### Fluke stroke rate

The full baseline model (B1; Table A1) for fluke stroke rate did not converge unless presence/absence of UPAS was removed as a covariate. There was clear support for the factor covariate state (d.f.=4, *F*=161, *P*<0.001) and time since solar noon (edf=2.8, *F*=3.6, *P*<0.001) in the full baseline model (B1; Table A1). No other covariates were supported at the 5% level. Therefore, only state and solarnoon were included in the final baseline model for fluke stroke rate. Virtually no serial correlation remained in the residuals of this model.

Sonar order effects were supported in all exposure models for fluke stroke rate that included them (E1–E3). There was no difference in support for SELmax_prev and SPLmax_prev (edf=0.81, *F*=1.1, *P*=0.020; model E3). In the exposure model, there was support for presence/absence of MPAS (d.f.=1, *F*=4.3, *t*=2.0, *P*=0.038). No other exposure effects, post-exposure effects or blackfish exposure effects were supported at the 5% level. Therefore, the final exposure model for fluke stroke rate included solarnoon, SELmax_prev and MPAS. Fluke stroke rates were estimated to decrease slightly during sonar exposures as a function of previous SEL exposure. Compared with CAS and HPAS, the rates were somewhat higher during MPAS exposures (Fig. S5). However, the model only explained ∼11% of the variation in the response data. The fit of the final exposure model for fluke stroke rate was assessed by plotting observed versus fitted values (Fig. S6).
