# Supplementary material for: When the noise goes on: received sound energy predicts sperm whale responses to both intermittent and continuous navy sonar
Source: J Exp Biol. 2020 Apr 6;223(7):jeb219741. doi: 10.1242/jeb.219741 (PMC7157582; doi:10.1242/jeb.219741)
Supplement: Supplementary information [file jexbio-223-219741-s1.pdf]

Supplementary figures

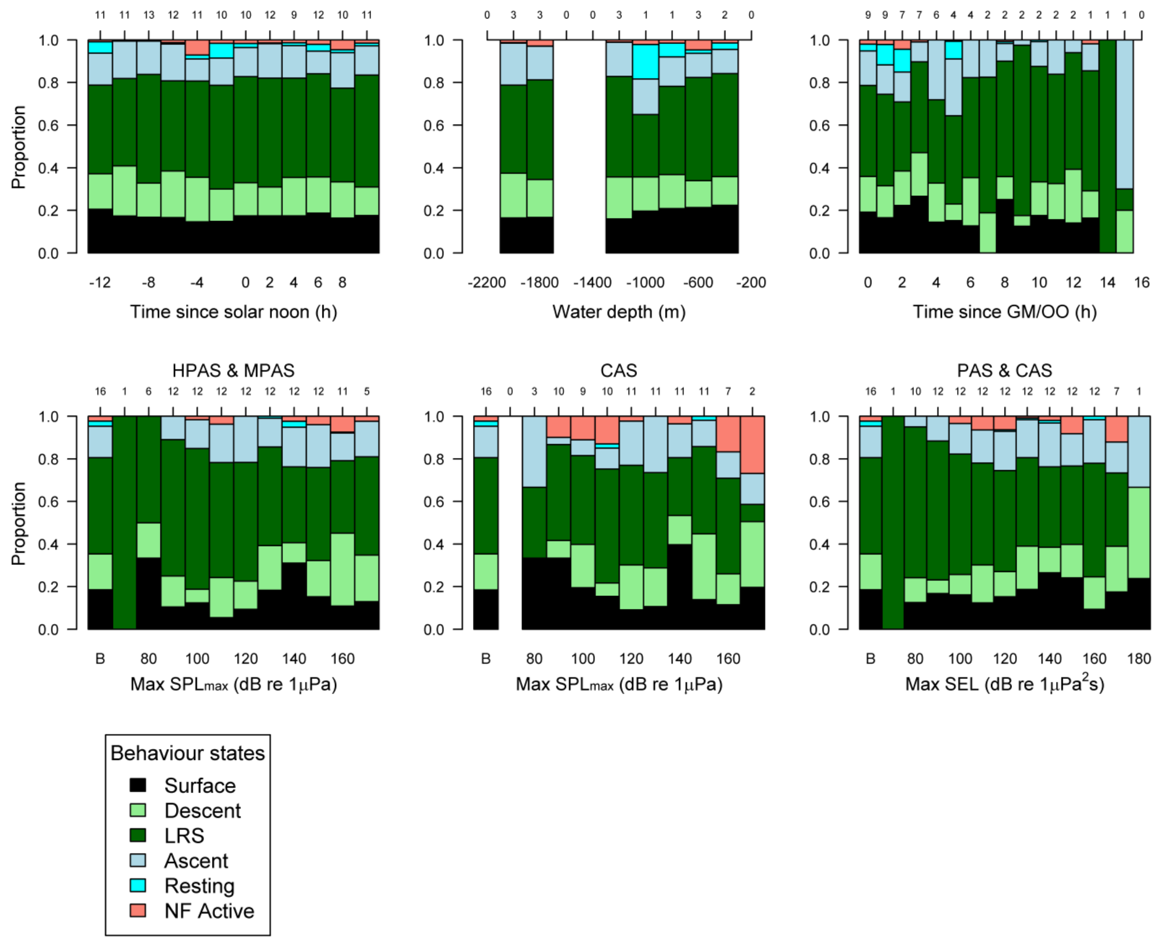

Figure S1. Individual-average time budgets during baseline (top panels, excluding 20-min post-exposure data) and during exposures (bottom panels). Top x-axis shows number of individuals used to calculate the average for each bin; bottom-axis 'B' indicates baseline.

Journal of Experimental Biology • Supplementary information

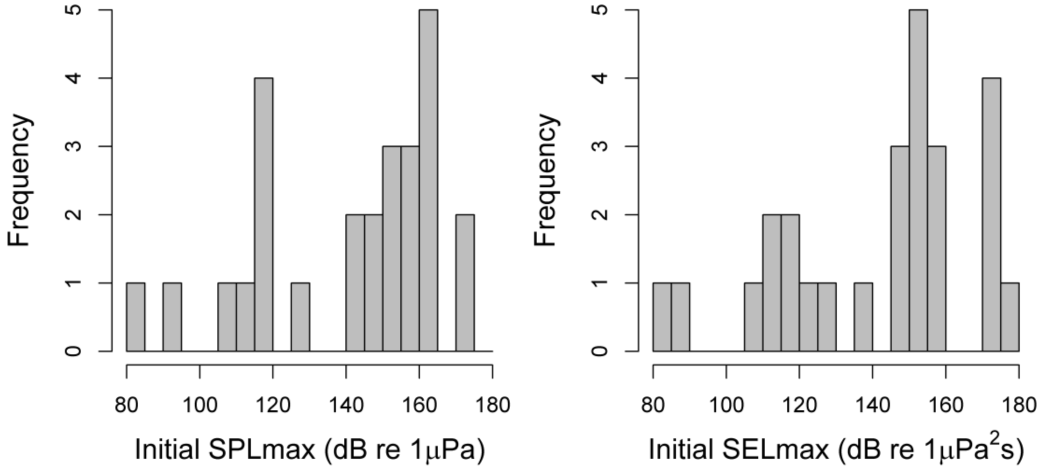

Figure S2. Fourteen animals switched to non-foraging active state following sound exposure (mean and median SELmax at onset 141 and 150 dB re 1 μPS2 s, respectively; n=26).

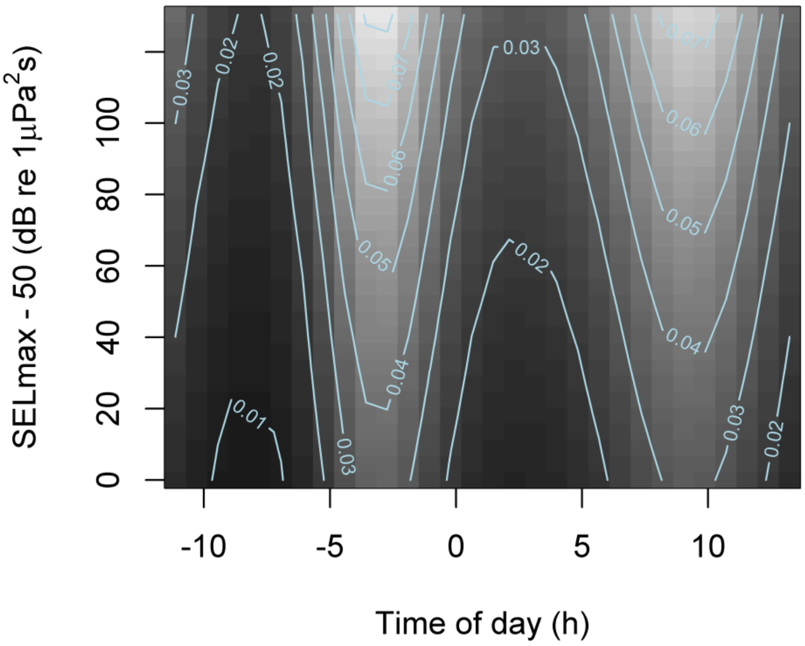

Fig. S3. Predictions from the final exposure model for non-foraging active state. Lighter colour indicates greater probability of non-foraging active state. SELmax values are shown as dB above the values assigned to non-exposure data (50 dB), which is how the covariate was encoded in the model.

Journal of Experimental Biology • Supplementary information

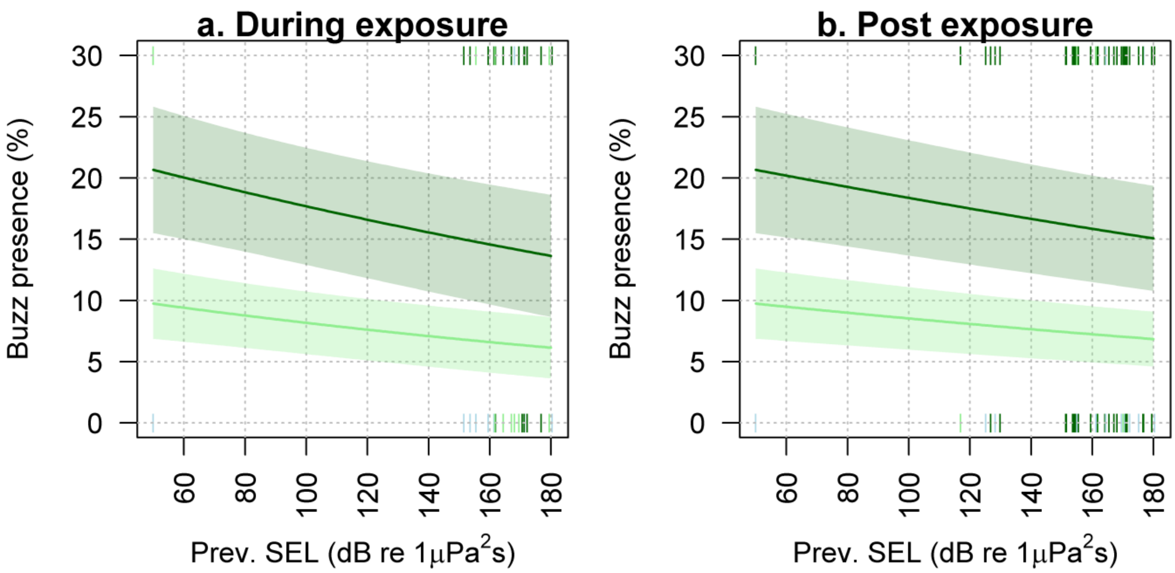

Fig. S4. Model predictions for buzz presence as a function of the maximum SEL exposure in the previous exposure session. For buzz presence, the effect of previous SEL exposure was supported for both subsequent exposures (panel a) and post-exposure periods (panel b). Model predictions for buzzing are shown separately for when the animal is in LRS (dark green), or non-LRS (light green) behaviour state, overlaid with data (shown as rug plot). Nevertheless, ROC analysis indicated relatively weak model fit.

Journal of Experimental Biology • Supplementary information

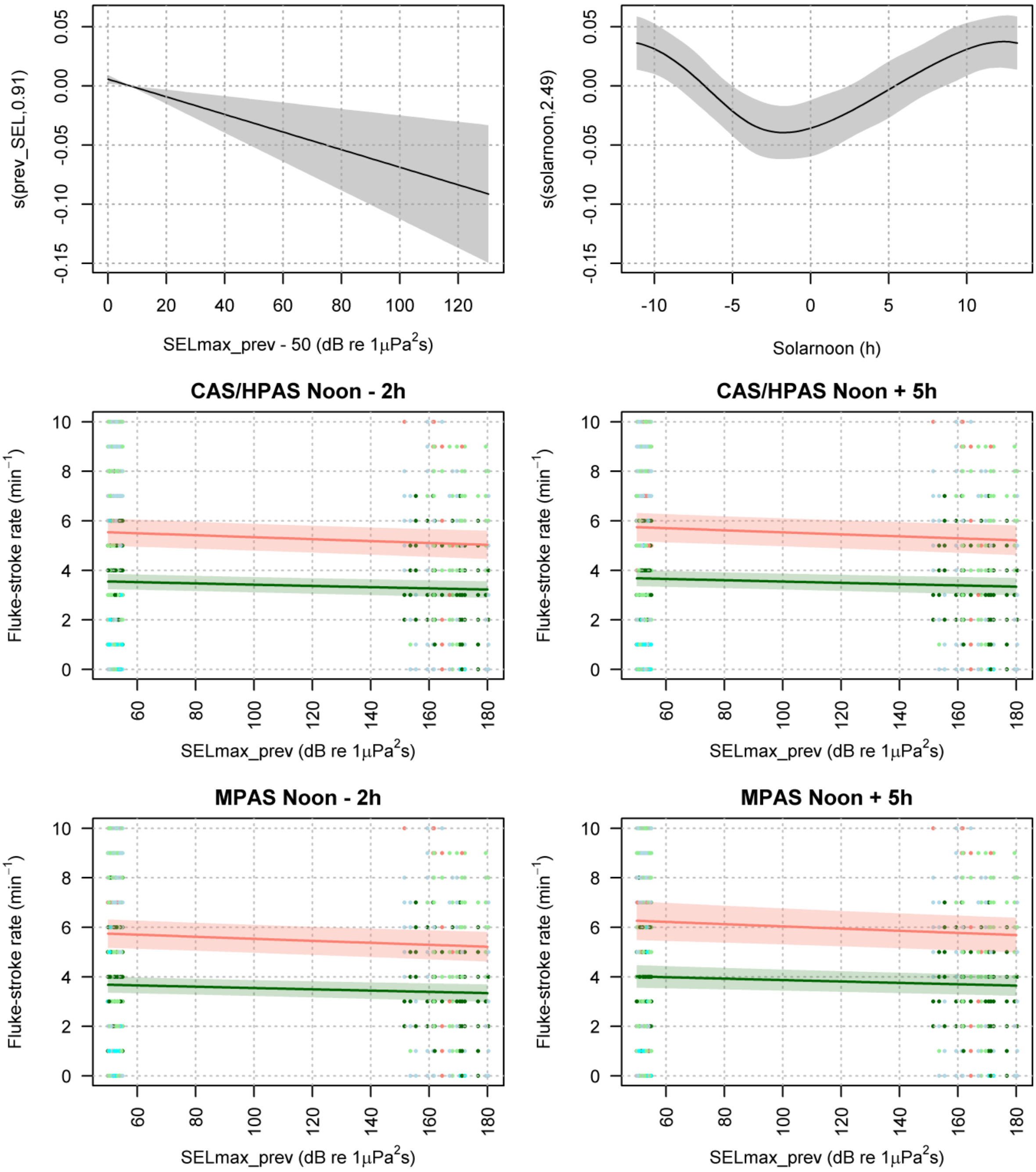

Fig. S5. Estimated effects on fluke-stroke rates. Top panels: component smooths at the scale of the linear predictor (function plot.gam output, library mgcv), middle and bottom panels: model predictions during sonar exposure as a function of maximum SEL achieved in previous sessions. Predictions are given for two different values for time since solar noon, and for MPAS vs. other exposures. Predictions are overlaid with data, colour-coded by the most likely state (random variation added to baseline values at x-direction). Salmon: non-foraging active state; light blue: ascent, light green: descent, dark green: LRS, cyan: resting/drifting

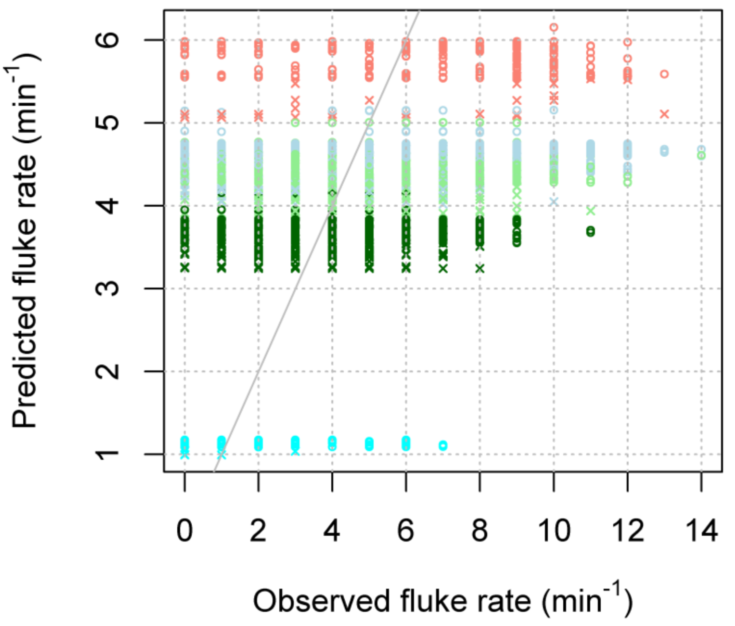

Fig. S6. Observed vs. predicted fluke-stroke rate from the selected exposure model (fl_rate ~ s(Solarnoon) + s(SELmax_prev) + MPAS). Colours indicate most likely behaviour state from the hidden state model (Salmon: non-foraging active state; light blue: ascent, light green: descent, dark green: LRS, cyan: resting/drifting).

Journal of Experimental Biology • Supplementary information
